# High-Content Analysis of 3D Chondrogenic Pellets Derived from Primary Cells In Vitro

**DOI:** 10.3390/biomedicines14071496

**Published:** 2026-07-01

**Authors:** Lucija Voga, Tilen Burnik, Maša Kandušer, Matjaž Jeras, Janja Zupan, Andreja Trojner Bregar

**Affiliations:** 1 Department of Clinical Biochemistry, Faculty of Pharmacy, University of Ljubljana, Aškerčeva 7, 1000 Ljubljana, Sloveniatilen.burnik@ffa.uni-lj.si (T.B.); matjaz.jeras@ffa.uni-lj.si (M.J.); janja.zupan@ffa.uni-lj.si (J.Z.); 2 Department of Biochemistry, Faculty of Chemistry and Chemical Technology, University of Ljubljana, Večna pot 113, 1000 Ljubljana, Slovenia; 3 Institute of Pharmacy, Faculty of Pharmacy, University of Ljubljana, Aškerčeva 7, 1000 Ljubljana, Slovenia; masa.kanduser@ffa.uni-lj.si; 4 Department of Perinatology, Division of Gynecology and Obstetrics, University Medical Centre Ljubljana, Šlajmerjeva 4, 1000 Ljubljana, Slovenia; 5 Faculty of Medicine, University of Ljubljana, Vrazov trg 2, 1000 Ljubljana, Slovenia

**Keywords:** primary MSCs, 3D chondrogenesis, pellets, high-content analysis, type II collagen, immunofluorescence

## Abstract

**Background**: Primary cells derived from connective tissues contain mesenchymal stem/stromal cell (MSC)–like progenitors with chondrogenic potential relevant for cartilage repair. However, donor- and tissue-specific variability and the lack of robust, high-content analytical methods limit their translational use. **Objectives**: This study aimed to develop and optimize a high-content imaging workflow for quantitative evaluation of chondrogenesis in three-dimensional (3D) pellets derived from primary cells. **Methods**: Primary human cells isolated from cartilage were chondrogenically differentiated in vitro. A systematic optimization of immunofluorescence staining parameters was performed, including staining platform, enzymatic matrix digestion, non-specific site blocking, membrane permeabilization, and nuclear counterstaining. Type II collagen was detected using an Alexa Fluor 488–conjugated antibody, and pellets were analyzed using high-content non-confocal imaging. Fluorescence intensities were adjusted to the pellet area to account for size-dependent effects. **Results**: Staining directly in imaging plates enabled streamlined high-content analysis. Controlled pepsin-mediated matrix digestion markedly enhanced antibody penetration, while excessive digestion compromised pellet integrity. Extended bovine serum albumin blocking improved type II collagen signal intensity and homogeneity. Triton X-100 permeabilization increased detection sensitivity but occasionally induced structural disruption in weakly organized control pellets. The optimized protocol enabled clear discrimination between chondrogenic pellets and controls, with approximately threefold higher type II collagen signal in chondrogenic samples. **Conclusions**: This study establishes a high-content imaging–based workflow for quantitative assessment of 3D chondrogenesis from primary cells. The approach provides a rapid, scalable platform with direct relevance for in vitro screening, potency testing, and quality control in cartilage-oriented advanced therapy development.

## 1. Introduction

Articular cartilage injuries and degenerative joint disorders represent a major clinical and socioeconomic challenge due to the inherently limited regenerative capacity of hyaline cartilage. Osteoarthritis (OA), the most prevalent degenerative joint disorder, continues to rise globally, with an estimated 607 million affected individuals and tens of millions of new diagnoses recorded in 2021 alone [1]. The increasing life expectancy and prevalence of obesity are expected to further accelerate this trend, contributing to a substantial burden on healthcare systems and compromised quality of life for aging populations [2].

Primary cells isolated from adult tissues—including bone, adipose tissue, muscle, synovium, and other connective tissues—that exhibit mesenchymal stem/stromal cell (MSC)-like characteristics hold considerable therapeutic promise for OA. Their regenerative capacity, coupled with their immunomodulatory functions, positions them as highly attractive candidates for OA treatment [3]. Building on these biological advantages, recent decades have witnessed the emergence of advanced therapy medicinal products (ATMPs) as a promising avenue for repairing focal cartilage defects and potentially altering disease progression. Autologous chondrocyte–based products such as Spherox^®^ (registered by the European Medicines Agency) and MACI^®^ (registered by the Food and Drug Administration) are clinically approved ATMPs that utilize primary human chondrocytes cultured ex vivo. Spherox^®^, comprising spherical aggregates of autologous chondrocytes embedded in their self-produced extracellular matrix, is indicated for treating full-thickness cartilage defects of the femoral condyle and patella (ICRS grade III–IV). MACI^®^ consists of autologous chondrocytes seeded onto a porcine collagen I/III membrane for treatment of symptomatic cartilage defects of the knee. Despite their clinical success, these ATMPs rely on harvesting and expanding autologous chondrocytes, a process that is frequently limited by donor age, tissue quality, and the intrinsic decline in cellular potency. Age-associated stem cell exhaustion—recognized as a hallmark of aging [4]—compromises not only tissue-resident stem cell pools but also their downstream chondrogenic progenitors [5]. Our previous work demonstrated that patients with OA exhibit diminished colony-forming efficiency and reduced chondrogenic capacity of bone- and muscle-derived mesenchymal stem/stromal cells (MSCs) compared to healthy controls [6,7]. These findings underscore important concerns regarding the suitability of autologous cell sources for therapeutic applications in individuals already affected by degenerative joint pathology. Moreover, they underscore the need for a rapid and reliable method to evaluate the chondrogenic capacity of patient-derived primary cells in vitro prior to their clinical use in cartilage regeneration.

Chondrogenic differentiation of primary cells in vitro can be induced under appropriate cues, including transforming growth factor (TGF)-β1/β3, insulin-like growth factor 1, dexamethasone, ascorbate, insulin, transferrin, and selenite supplement [3]. However, traditional two-dimensional (2D) monolayer culture systems, despite their widespread use, do not recapitulate the native three-dimensional (3D) extracellular matrix (ECM)–rich microenvironment that is essential for promoting and stabilizing chondrogenic lineage commitment. In contrast, 3D culture models such as pellets, hanging drops, organoids, and spheroids offer more physiologically relevant systems that enhance cell–cell interactions, ECM deposition, and lineage-specific differentiation. Pellets in particular provide controllable, scaffold-free microtissues that mimic aspects of native tissue physiology and have become a central tool for engineering complex 3D tissues [8]. These features make 3D models indispensable for studying cartilage formation, evaluating novel regenerative strategies, and developing ATMPs.

Despite advances in 3D culture systems, the field lacks standardized, robust, and high-content in vitro assays capable of reliably evaluating the quality of cartilage-like tissue formed by primary cells or MSCs. This gap represents a major bottleneck in ATMP development, where regulatory frameworks require stringent and reproducible potency and quality assessments. Current analytical approaches—such as histology, biochemical ECM quantification, mechanical testing, and gene expression profiling—are often destructive, low-throughput, labor-intensive, and highly operator-dependent. Based on these limitations, there is an urgent need for fast, high-content, and reproducible 3D tissue analytics.

Furthermore, conventional methods struggle to capture pellet heterogeneity and dynamic ECM deposition over time, parameters that are critical for ensuring batch comparability and predicting clinical outcomes in ATMP manufacturing. High-content imaging and automated image analysis offer a promising solution; however, validated protocols for the analysis of 3D chondrogenesis remain scarce.

The objective of this study was to develop and optimize a high-content imaging workflow for evaluating type II collagen–rich neocartilage formation in chondrogenic pellets generated from primary somatic cells or MSCs. Following standardized culture conditions for pellet formation, we have optimized immunofluorescence staining for type II collagen and implemented an automated image analysis pipeline for quantitative assessment. The resulting method enables efficient evaluation of multiple pellets simultaneously, while requiring minimal cell numbers, and offers a scalable and reproducible platform for assessing chondrogenic capacity in vitro. The high-content analysis thus provides a possible solution to a critical methodological gap in ATMP development by providing a rapid and quantitative approach for assessing cartilage quality during early-stage screening and process development.

## 2. Materials and Methods

### 2.1. Human Tissue Samples

Human articular cartilage was obtained from three donors (donor A, female, 53 years; donor B, female, 60 years; and donor C, female, 74 years) undergoing routine total or partial knee arthroplasty at the Department of Orthopaedic Surgery at the University Medical Centre Maribor. Approval for this study was obtained from the National Medical Ethics Committee of the Republic of Slovenia (reference number: 0120-268/2020/3). Written informed consent was obtained from all the patients included in the study. Primary MSCs from two cadaveric donors (donor D, female, 65 years; donor E, male, 32 years) were used from our previous study [9]. Briefly, primary cells were isolated from trabecular bone marrow and periosteum, and their MSC-like properties were demonstrated [9]. Approval for this study was obtained from the National Medical Ethics Committee of the Republic of Slovenia (reference number: 0120-523/2016/15).

### 2.2. Primary Human Cell Culture

Cartilage explants were transferred to low-glucose Dulbecco’s modified Eagle’s medium (LG-DMEM; Biowest, Nuaillé, France), supplemented with 1% glutamine and 2% penicillin and streptomycin (all Biowest), without serum and processed at room temperature within 24 h. All isolation procedures and in vitro experiments were performed at the Faculty of Pharmacy, University of Ljubljana. Primary chondrocytes were isolated following a previously described protocol [10]. Briefly, cartilage biopsies (approximately 1–2 cm^3^) were minced and digested in LG-DMEM containing 1 mg/mL collagenase from Clostridium histolyticum (C1639, Sigma-Aldrich, Burlington, MA, USA) at 37 °C for 24 h with constant agitation. Isolated cellular fractions were seeded at passage (p) 0 in T175 flasks using LG-DMEM supplemented with 10% fetal bovine serum (Gibco, Grand Island, NY, USA, Cat. No. 10270106 in LOT 42F9681K), 1% glutamine, and 2% penicillin and streptomycin (all Biowest). Once cultures reached 80–90% confluence, the cells were detached with trypsin-EDTA and cryopreserved in FBS with 10% dimethyl sulfoxide. Cryopreserved cells were later thawed and expanded in culture to obtain sufficient quantities appropriate for optimization of the current analysis. Primary bone marrow (donor D)- and periosteum (donor C)-derived MSCs from our previous study [9] were thawed and subsequently expanded in culture to obtain sufficient cell numbers for validation of the optimized protocol. Human fibroblast cell line BJ-5ta (ATTC, Nuth, The Netherlands, CRL-4001) was used as a negative cell control (donor F) in the final optimized protocol. Cell morphology and confluence were monitored and imaged using Evos XL inverted microscope (LifeTechnologies, Carlsbad, CA, USA).

### 2.3. Chondrogenic Differentiation

Chondrogenic differentiation in vitro was performed as previously described [6,7,9,11]. Briefly, pellets were created by centrifuging 100.000 cells per pellet at 300× *g* for 10 min. Primary chondrocytes from donor A were passage (p) 4, from donor B, p4 and p5, and from donor C, p4. Primary MSCs from donors D and E were p3. Fibroblasts BJ-5ta (donor F) were p22. The resulting cell pellets were incubated overnight in 15-mL conical tubes at 37 °C in a humidified atmosphere containing 5% CO_2_ to allow 3D pellet formation. The following day, the supernatant was carefully removed and replaced in treated pellets with 500 μL of chondrogenic differentiation medium composed of high-glucose DMEM (Biowest), 100 nM dexamethasone (Sigma, Kawasaki, Japan), 50 µg/mL 2-phospho-L-ascorbic acid trisodium salt (Sigma), 1% insulin-transferrin-selenium (Sigma), 10 ng/mL TGF-β1 (Gibco), and antibiotic-antimycotic solution (Biowest). On the other hand, control pellets received 500 μL of non-differentiation medium, containing the same components as chondrogenic differentiation medium except for the chondrogenic inducer TGF-β1 (no-TGF-β1 controls). Pellets were cultured for 21 days, with media changes performed every two to three days.

### 2.4. Optimizing Immunofluorescence Staining Protocol

To optimize the immunofluorescence staining protocol, pellets were collected from the culture medium and washed with PBS. A subset of samples was transferred to 96-well Revvity CellCarrier Spheroid ULA plates (Revvity, Waltham MA, USA) for staining and imaging, while the remaining pellets were kept in 15-mL conical tubes to evaluate an alternative staining platform. Pellets were fixed in 200 μL of 10% neutral buffered formalin (Sigma-Aldrich) for 10 min at room temperature, followed by two washes with Dulbecco’s PBS (Sigma-Aldrich). To enable controlled antigen retrieval, samples were equilibrated in 200 μL of 0.2 M HCl (Sigma) for 5 min at 37 °C, followed by incubation with 0.5 g/L pepsin (Roche, Basel, Switzerland, Cat. No. 10108057001, ~2500 units/mg protein) in 0.2 M HCl for 10–30 min at 37 °C. Digestion was stopped by incubation in 200 μL of Milli-Q water for 5 min at room temperature, followed by a PBS wash. Cell membranes were permeabilized three times with 200 μL of 0.2% Triton X-100 (Sigma) for 5 min at room temperature. Non-specific binding sites were then blocked with 200 μL of 1% bovine serum albumin (BSA, Sigma-Aldrich) in 0.2% Triton X-100 for 30–45 min at room temperature, followed by a PBS wash. Pellets were incubated with 200 μL of anti-type II collagen antibody conjugated with Alexa Fluor (AF) 488 (SouthernBiotech, Birmingham, AL, USA), diluted 1:100 in PBS, for 24 h at 2–8 °C in the dark. After primary antibody incubation, samples were washed twice with PBS and once with 0.2% Triton X-100 in PBS for 10 min at room temperature. Nuclear staining was performed using 200 μL of Hoechst 33342 (Thermo Fisher, Waltham, MA, USA), diluted 1:2000 in PBS, for 20 min or 1 h at room temperature in the dark, followed by two PBS washes. Pellets initially kept in 15-mL conical tubes were transferred to imaging plates before acquisition. For one sample, Triton X-100 was omitted, and all corresponding steps were performed using PBS.

### 2.5. High-Content Imaging Acquisition and Analysis

High-content imaging was performed using the Revvity Operetta^®^ CLS™ system controlled by Harmony™ software (version 5.2.2). Pellets were imaged in 96-well Revvity CellCarrier Spheroid ULA plates using a 10× air objective (NA 0.3) in a non-confocal mode. Acquisition channels were configured to sequentially capture AF 488, Hoechst 33342, and brightfield signals. AF 488 was detected using 460–490 nm excitation and 500–550 nm emission, with an exposure time of 15 ms and 33 mW illumination power. Hoechst 33342 was acquired using 355–385 nm excitation and 430–500 nm emission, with an exposure time of 5 ms and 5.2 mW illumination power. Brightfield images were acquired using an exposure time of 6 ms and 4 mW illumination power. These parameters were set in a way to minimize photobleaching and to maximize the use of the detector’s dynamic range. Z-stacks were acquired from the bottom of the well with a step size of 20.0 µm across 15 planes. The acquired Z-stacks covered only the optically accessible portion, i.e., outer and intermediate regions of the pellets, rather than their full physical volume, to capture regions with sufficient signal quality and reproducibility.

Image analysis was performed using Harmony™ software by defining an automated analysis sequence. All three acquisition channels were selected as the input for all processing steps. Maximum intensity projection was applied to the acquired Z-stacks to generate a single global image per sample. The pellet’s region was identified using the Find Image Region function based on the AF 488 signal intensity, which was present throughout the ECM. The region was segmented using a lower intensity threshold applied to the AF 488 channel. Brightfield images were used as a morphological reference but were not used for segmentation due to heterogeneous intensity distribution within pellets. Within the region, a lower intensity threshold was applied to remove background fluorescence, with no upper limit. Object splitting was enabled to separate distinct regions where applicable. Fluorescence quantification for both dyes was performed using the Calculate Intensity Properties function applied to the identified region. Extracted parameters included mean intensity, standard deviation, and coefficient of variation. Pellet cross-sectional area was quantified using the Calculate Morphology Properties module on the resulting global image. All parameters, along with the number of detected objects, were compiled using the *Define Results* step to generate a complete output dataset.

### 2.6. Statistical Analysis

**Figure 1:**
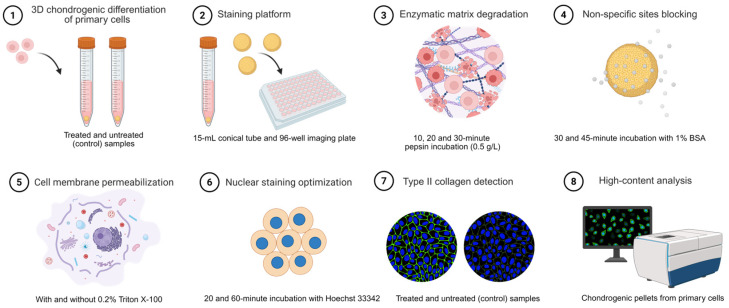
The schematic representation of the study. Created in BioRender. Zupan, J. (2026). https://BioRender.com/gv2ece0 (accessed on 12 June 2026).

Global intensities for AF 488 and Hoechst 33342, together with the corresponding pellet region of interest (ROI) area, were used to calculate size-adjusted intensities according to the following formula: (AF 488 or Hoechst 33342 intensity) × (pellet ROI area). This normalization was applied to account for variability in pellet size, which can influence staining intensity due to differences in dye distribution. Specifically, larger pellets tend to exhibit lower signal intensity per unit area, reflecting an inverse relationship between pellet size and measured fluorescence. Normalization was based on the 2D ROI area rather than pellet volume, due to technical limitations in Z-stack acquisition that precluded full volumetric imaging of the pellets. The results are expressed as the mean intensity ± standard deviation (SD) and coefficient of variation (CV). These parameters were calculated automatically by the image analysis software and reflect variations in pixel intensities within a single analyzed pellet image. The schematic representation of the study is shown in Figure 1.

## 3. Results

### 3.1. Experimental Design and Immunostaining Parameters

To adapt immunofluorescence protocols from conventional 2D cultures to 3D models, we systematically optimized multiple staining parameters, in particular the staining platform, enzymatic matrix degradation, non-specific sites blocking, cell membrane permeabilization, nuclear staining, and type II collagen detection. An overview of the experimental design, including sample identifiers, is provided in Table 1.

A range of staining platforms, fixation, blocking, permeabilization, and nuclear counterstaining conditions were evaluated to determine their effects on staining quality and signal acquisition. Key variables included the presence or absence of pepsin digestion, BSA-based blocking, Triton X-100 permeabilization, and variations in incubation times. This systematic assessment allowed the identification of staining parameters compatible with the structural complexity of 3D tissue models.

### 3.2. Staining Platform

**Figure 2:**
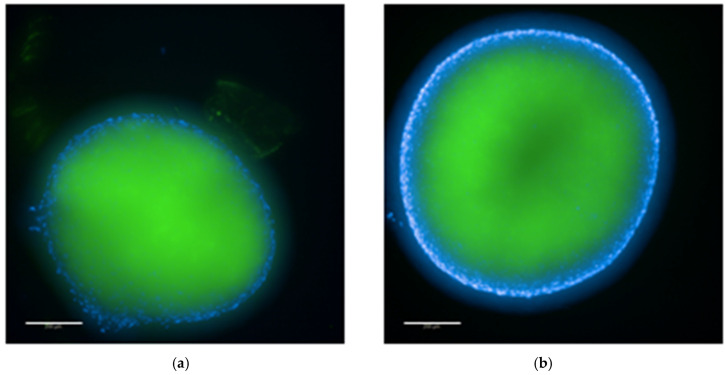
Comparison of staining platforms. Sample 3 was stained in a 15-mL conical tube (**a**), and sample 9 in a 96-well imaging plate (**b**). In both samples, the type II collagen (AF 488, green) signal exhibited a comparable distribution pattern, with distinct nuclei (Hoechst, blue). The sample in (**a**) shows slightly irregular morphology with a visible adhesion site on the lower left side of the pellet. Images were acquired and displayed using identical settings. The scale bar represents 200 µm.

To identify the optimal staining platform for high-content analysis of 3D chondrogenesis, samples were processed either in 15-mL conical tubes or directly in a 96-well imaging plate. Representative fluorescence images of each platform illustrating signal distribution and dye penetration for the pellets are shown in Figure 2.

Quantitative fluorescence intensity measurements for AF 488 are summarized in Table 2 and for Hoechst 33342 in Supplementary Table S1.

**Figure 3:**
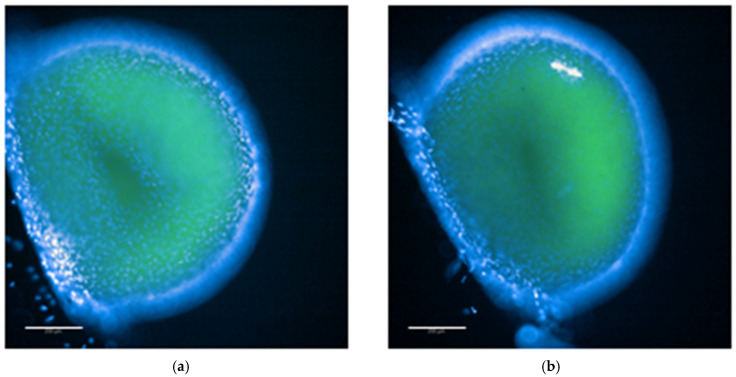
Morphological alterations of pellets stained in 15-mL conical tubes. Representative image of pellets, samples 4 (**a**) and 5 (**b**), processed in 15-mL conical tubes. These pellets exhibit a distinct single adhesion point at the site of contact with the tube wall, producing localized irregularity within an otherwise spherical structure. Images were acquired and displayed using identical settings. The scale bar represents 200 µm.

Mean and variability of AF 488 fluorescence intensity were higher when pellets were processed directly in 96-well imaging plates than in 15-mL conical tubes. Also, Hoechst 33342 intensity was higher in the imaging plate than in the conical tube, albeit with greater variability (Supplementary Table S1). Notably, pellets stained in conical tubes often exhibited a visible attachment point to the plastic wall, leading to more irregular and distorted morphologies within an otherwise spherical structure (Figure 3). In contrast, staining directly in imaging plates better preserved their rounded shape.

### 3.3. Enzymatic Matrix Degradation

Pepsin was selected for enzymatic matrix degradation. Three pepsin incubation times were evaluated to determine the optimal antigen retrieval and matrix degradation enabling maximal penetration of AF 488-conjugated anti-collagen II antibodies into the pellet core. Incubation durations of 10, 20, and 30 min were tested, along with no-pepsin controls, one without (sample 1) and one with blocking non-specific binding sites (sample 2). The quantitative results are summarized in Table 3 for AF 488 and in Supplementary Table S2 for Hoechst 33342.

**Figure 4:**
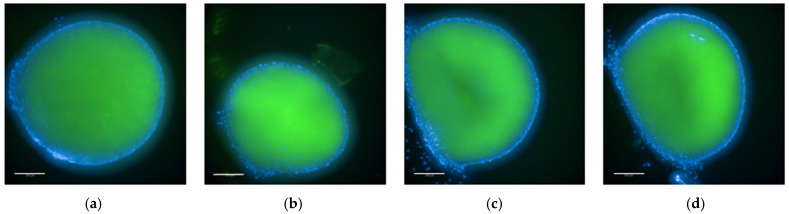
Comparison of pellet morphology following different durations of pepsin digestion. Sample 2 (**a**) was not exposed to pepsin, whereas sample 3 (**b**) was pepsin-treated for 10 min, sample 4 (**c**) for 20 min, and sample 5 (**d**) for 30 min. Sample 3 (**b**) exhibits a visibly smaller pellet diameter compared with the other three samples. In samples 4 (**c**) and 5 (**d**), partial detachment of peripheral cells is evident, predominantly on the side that had been in contact with the plastic tube during incubation. All images were acquired and displayed using identical settings. The scale bar represents 200 µm.

Diffusion-dependent steps in the staining protocol, including pepsin incubation, are strongly influenced by pellet size. This is the reason we excluded the smallest sample, i.e., sample 3, from the analysis. Its exclusion was primarily justified by the likelihood that pepsin would penetrate more rapidly and extensively into the smaller pellets during the fixed incubation time, resulting in disproportionately greater matrix digestion relative to larger pellets. Sample 2, which did not undergo pepsin treatment, also differed in pellet size. However, because it was not subjected to pepsin-mediated degradation, it was not excluded from the analysis. Therefore, among the four remaining pellets, clear differences were observed between conditions with and without pepsin treatment. The intensity of the AF 488 signal was consistently higher in pellets treated with pepsin compared to those without treatment. Visual inspection further indicated that a 30-min incubation induced detachment of some peripheral cells, suggesting over-digestion at longer incubation times (Figure 4). We emphasize that the reported digestion times (10–30 min) should be regarded as guideline ranges rather than fixed parameters, as batch-specific optimization may be necessary, particularly when using enzymes with varying activity profiles.

### 3.4. Non-Specific Sites Blocking

To determine the optimal duration for blocking non-specific binding sites, two BSA incubation times, i.e., 30 and 45 min, were tested. Samples 11–14 were subjected to an extended Hoechst 33342 staining step (60 min), whereas samples 10 and 16 were processed using a 20-min nuclear staining procedure. Consequently, comparisons were made only between samples processed under identical staining conditions. A summary of quantitative results is presented in Table 4.

**Figure 5:**
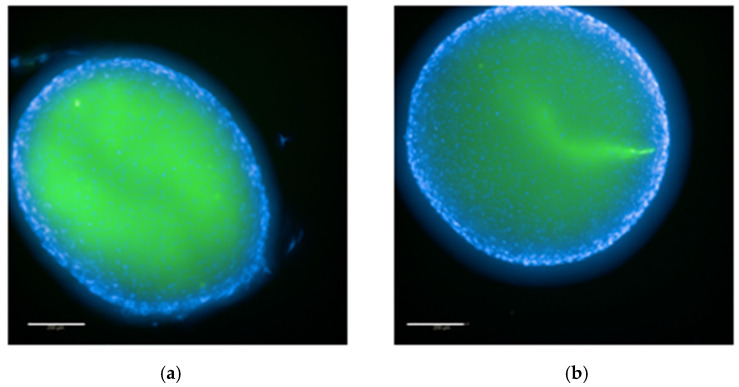
Comparison of different BSA incubation times. (**a**) Sample 16 with 45 min BSA incubation time and (**b**) sample 10 with 30 min BSA incubation time. Images were acquired and displayed using identical settings. The scale bar represents 200 µm.

Under standard nuclear staining conditions, shortening the BSA incubation from 45 min (sample 16, Figure 5a) to 30 min (sample 10, Figure 5b) led to a reduction in mean AF 488 intensity, whereas Hoechst 33342 intensity remained comparable between the two conditions (Supplementary Table S3). The coefficient of variation (CV) for AF 488 was similar between the two conditions.

**Figure 6:**
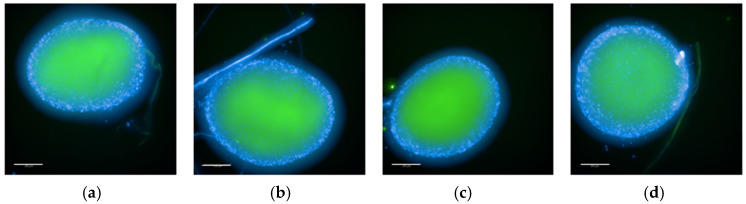
Comparison of different BSA incubation times with an extended Hoechst 33342 incubation time. Samples 11 (**a**) and 12 (**b**) with 45 min BSA incubation time. Samples 13 (**c**) and 14 (**d**) with 30 min BSA incubation time. Images were acquired and displayed using identical settings. The scale bar represents 200 µm.

Within the extended nuclear staining set, the pellets incubated with BSA for 45 min (samples 11 and 13, Figure 6a,b) and those incubated for 30 min (samples 13 and 14, Figure 6c,d) showed no difference in AF 488 mean intensities. The 45-min incubation was associated with lower CV of AF 488 intensity compared with the 30-min incubation time, indicating more homogeneous staining. Hoechst 33342 intensities did not vary with BSA incubation time across the samples (Supplementary Table S3).

### 3.5. Cell Membrane Permeabilization

To assess whether the protocol could be optimized to better preserve pellet morphology while eliminating unnecessary chemical exposure, Triton X-100 was omitted in one staining condition (sample 15), where it was excluded from all steps and replaced with PBS. All the other steps were carried out under standard staining conditions (Table 5) in the same manner as with the sample, including Triton X-100 (sample 16).

**Figure 7:**
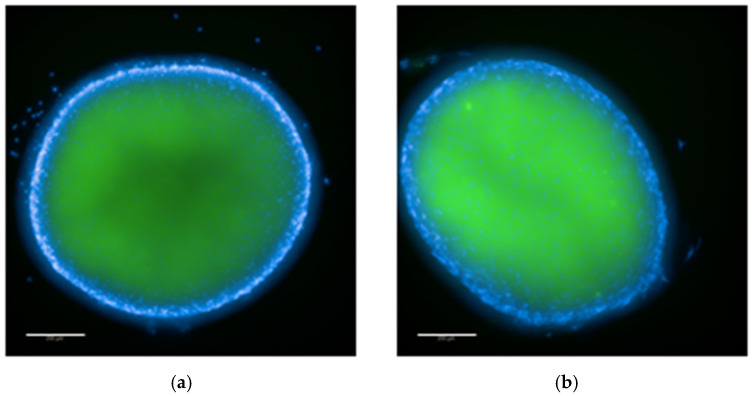
Effect of Triton X-100 on fluorescence staining. Representative images obtained using a staining protocol excluding (**a**) or including Triton X-100 (**b**). Images were acquired and displayed using identical settings. The scale bar represents 200 µm.

The exclusion of Triton X-100 from the staining protocol resulted in a marked reduction in fluorescence intensity for both AF 488 and Hoechst 33342 (Figure 7), indicating decreased staining efficacy under non-permeabilizing conditions.

### 3.6. Nuclear Staining

**Figure 8:**
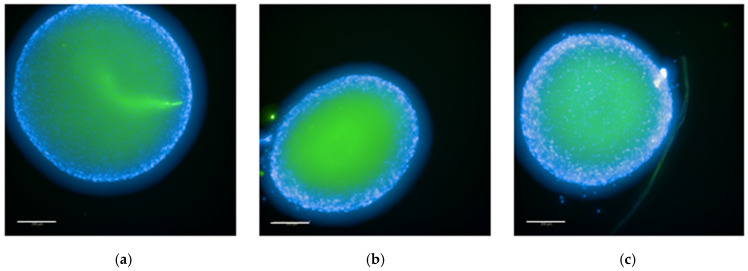
Comparison of different Hoechst 33342 incubation times following a 30-min BSA incubation. Sample 10 (**a**) was incubated with Hoechst 33342 for 20 min, whereas samples 13 (**b**) and 14 (**c**) were incubated for 60 min. Images were acquired and displayed using identical settings. The scale bar represents 200 µm.

**Figure 9:**
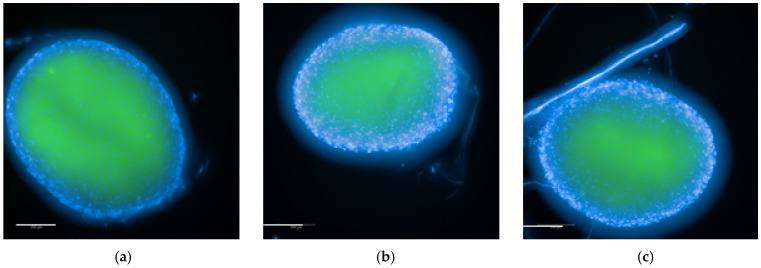
Comparison of different Hoechst 33342 incubation times following a 45-min BSA incubation. Sample 16 (**a**) was incubated with Hoechst 33342 for 20 min, whereas samples 11 (**b**) and 12 (**c**) were incubated for 60 min. Images were acquired and displayed using identical settings. The scale bar represents 200 µm.

To determine whether prolonged incubation with Hoechst 33342 enhances nuclear staining within the pellet core, incubation periods of 20 and 60 min were compared. The analysis was performed across two independent experimental sets. In the first one, sample 8 (20-min incubation, Figure 8a) was compared exclusively with samples 13 and 14 (60-min incubation, Figure 8b,c). In the second set, sample 16 (20-min incubation, Figure 9a) was compared exclusively with samples 11 and 12 (60-min incubation, Figure 9b,c). Cross comparisons between the two experimental sets were not performed, as the pellets were subjected to different staining procedures.

Increasing the incubation time with Hoechst 33342 did not affect the AF 488 intensity. Therefore, this parameter was not considered relevant for further analysis. Since the fluorescence intensity of Hoechst 33342 can scale with pellet size due to dye concentration within the spheroid, size-adjusted intensities were compared (Table 6).

Although extended incubation resulted in higher raw Hoechst 33342 fluorescence, size-adjusted intensities indicated that this increase was primarily driven by differences in pellet size rather than improved nuclear staining within the pellet core under the tested conditions.

### 3.7. Type II Collagen Detection

**Figure 10:**
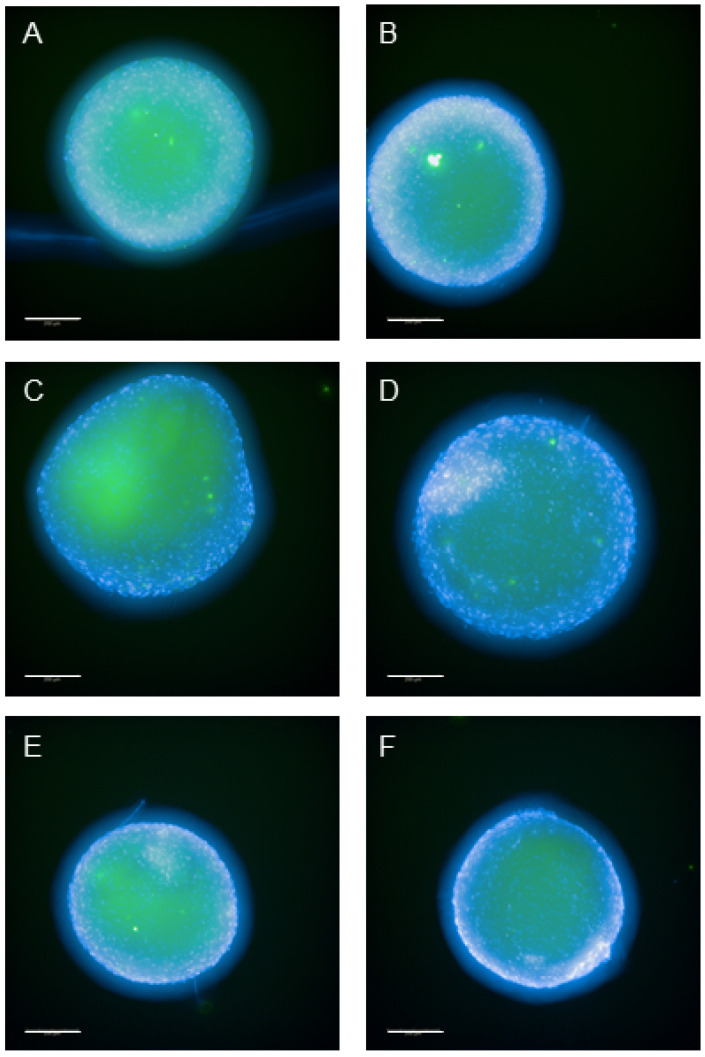
Comparison between samples undergoing chondrogenic differentiation (**A**,**C**,**E**) and no-TGF-β1 control (**B**,**D**,**F**). Representative images of pellets from primary chondrocytes (donor B), from primary bone-marrow-derived MSCs (donor E), and from fibroblast cell line BJ-5ta (donor F). Images were acquired and displayed using identical settings per donor pair. The scale bar represents 200 µm.

To assess the effectiveness of the optimized protocol to detect type II collagen, a main component of the hyaline cartilage, samples treated with chondrogenic media were compared with no-TGF-β1 controls (Figure 10).

During the staining protocol, one control sample (C3) was separated into two fragments, an event that occurred during the Triton X-100 permeabilization step. Despite this fragmentation, the sample was subsequently processed according to the original protocol without modifications, as the overall integrity of the fragments remained sufficient for downstream analysis. No fragmentation was observed with the rest of the nine control samples.

The results of this comparison are summarized in Table 7 for AF 488 and in Supplementary Table S5 for Hoechst 33342. To assess the potential contribution of autofluorescence to the AF 488 channel, samples 6, 7, 8, C1, and C2 were not stained with the type II collagen antibody. Sample 6 was fixed but not subjected to any step of the staining protocol (Table 7). Samples 7 and C1 were omitted from staining with the type II collagen antibody and Hoechst 33342, whereas all remaining protocol steps were carried out. Samples 8 and C2 were omitted from staining with the type II collagen antibody only, while all other steps of the protocol were completed. Although sample 6 exhibited relatively high AF 488 signal, indicating substantial background fluorescence in this channel, other samples (7, 8, C1, and C2) did not. Thus, the observed signal may be largely attributable to the fixation step alone. Furthermore, progression through the subsequent staining protocol appeared to reduce the background fluorescence, suggesting that the fixation-induced signal was at least partially diminished during later processing steps.

Fibroblasts were also included as a negative control for type II collagen antibody binding, as they are not expected to produce type II collagen. Although a slight difference in AF 488 fluorescence intensity was observed between the treated and no-TGF-β1 control fibroblast samples, both signals remained within the range of the background fluorescence previously observed in samples 7, 8, C1, and C2.

However, when the AF 488 intensity of treated samples was compared with that of the corresponding no-TGF-β1 controls, a generally higher AF 488 signal was observed in the treated samples. This trend was evident in chondrocytes (samples 16 vs. C3, 17 and 18 vs. C4 and C5, and 19 and 20 vs. C6 and C7), as well as in MSCs (samples 21 and 22 vs. C8 and C9, and 23 and 24 vs. C8 and C9). Nevertheless, some exceptions were noted, particularly among the chondrocyte samples (sample 18 vs. C4, and 20 vs. C6 and C7). Similar AF 488 signal intensity between the no-TGF-β1 control and the treated pellet was also observed in one sample of primary MSCs (donor D) (Table 7).

## 4. Discussion

Articular cartilage injuries and OA present a major clinical challenge due to the limited regenerative capacity of hyaline cartilage and the declining potency of autologous cell sources in affected and aging patients. Although 3D chondrogenic culture systems are increasingly used to model cartilage formation and support ATMP development, there is a lack of standardized, robust, and high-content in vitro assays for reliably assessing cartilage-like tissue quality. In the current study, we developed and optimized a fast, reproducible, high-content imaging–based workflow to quantitatively evaluate type II collagen–rich neocartilage formation in chondrogenic pellets derived from primary cells. We systematically evaluated several experimental parameters (Figure 1), including the staining platform (15-mL conical tube versus 96-well imaging plate), enzymatic matrix degradation (no pepsin versus varying pepsin incubation times), BSA blocking (no BSA versus different incubation times), permeabilization (absence or presence of Triton X-100), nuclear staining (absence or presence of Hoechst 33342), and the capacity to detect type II collagen (chondrogenically differentiated pellets versus control samples). The readouts for all parameters tested were AF 488 and Hoechst 33342 fluorescence intensities, adjusted to pellet area. Diffusion and enzymatic penetration strongly depend on pellet size, as smaller pellets possess shorter diffusion distances and a higher surface-area-to-volume ratio, rendering them more susceptible to rapid and extensive enzymatic degradation under identical incubation conditions. As previously reported, this size dependency can artificially enhance staining intensity in smaller pellets relative to larger ones, potentially confounding comparative analyses if pellet size is not controlled or accounted for during data interpretation [12,13]. Based on these considerations, we used size-adjusted fluorescence intensities in our study.

Our results showed that the first parameter, i.e., the choice of staining platform, had an effect on signal intensity, as the 96-well imaging plate platform produced higher fluorescence outcomes. Notably, pellets stained in conical tubes often exhibited a visible attachment point to the plastic wall, leading to more irregular and distorted morphologies within an otherwise spherical structure. In contrast, staining directly in imaging plates better preserved their rounded shape. However, despite these morphological irregularities observed in conical tubes, this effect was not considered critical and did not strongly impact our decision-making process. 96-well imaging plates nonetheless offered a clear practical advantage, including improved handling, compatibility with high-content imaging and analysis, and better preservation of pellet morphology. Consequently, 96-well imaging plates were selected for subsequent experiments.

The second evaluated parameter, i.e., the enzymatic matrix digestion, however, affected AF 488 signal intensity. It is well-recognized that efficient penetration of staining reagents into chondrogenic pellets remains a persistent technical challenge due to the dense ECM and tightly packed cellular architecture that characterize these 3D structures. In particular, cartilaginous pellets exhibit high concentrations of proteoglycans and collagen, which substantially restrict the diffusion of large macromolecules such as antibodies (~150 kDa), effectively creating a diffusion barrier within the pellet interior [13,14]. Importantly, the improved antibody signal observed following pepsin treatment suggests that insufficient matrix digestion may lead to underestimation of protein expression in pellet cores, a limitation that is often overlooked in qualitative and quantitative immunostaining analyses of 3D models. Enzymatic digestion of ECM components is therefore frequently required to enable deeper penetration and achieve homogeneous antibody labeling throughout the pellet volume. Our results show that the inclusion of a pepsin digestion step (both 20- and 30-min) enhances the penetration of AF 488–conjugated anti-type II collagen antibody into chondrogenic pellets. This observation aligns well with prior studies showing that targeted enzymatic degradation of ECM components can markedly improve macromolecular diffusion in 3D culture systems, resulting in more uniform and representative staining patterns [12,14,15]. Because enzymatic penetration depends strongly on pellet size, smaller pellets can undergo more extensive digestion within the same incubation time, which may artificially enhance staining intensity [12,13]. No differences were observed in samples without matrix degradation, regardless of BSA blocking of non-specific sites. Together, these findings suggest that matrix degradation is a critical prerequisite for efficient antibody binding, in agreement with earlier studies [12,14,15].

In contrast to antibodies, small-molecule-weight dyes such as Hoechst 33342 (616 Da) can readily diffuse through multiple cellular layers and ECM-rich regions with minimal impedance. Previous studies have demonstrated that DNA-binding dyes exhibit rapid and largely matrix-independent penetration kinetics, even in highly compact pellets, owing to their small size and favorable diffusion properties [14,16]. Consistent with previous reports, the comparable Hoechst 33342 fluorescence intensities observed across all experimental conditions in our study confirm that nuclear staining with this dye is not strongly dependent on pepsin matrix digestion.

However, the duration of enzymatic digestion can also critically influence the preservation of the pellet morphology. While enzymatic treatment can enhance antibody penetration by degrading ECM components, excessive digestion may compromise structural integrity. In the present study, different pepsin incubation times were systematically evaluated to determine an optimal balance between sufficient ECM permeabilization and maintenance of pellet morphology. However, visual inspection revealed that the 30-min treatment resulted in partial detachment of peripheral cells, indicating excessive matrix degradation and potential structural damage to the pellet. Considering both staining efficiency and preservation of pellet structure, the 20-min pepsin incubation represented the optimal condition in our experimental setup. These findings highlight the importance of carefully optimizing enzymatic digestion steps in 3D staining protocols, as both insufficient and excessive matrix degradation can negatively affect experimental outcomes [13].

The third parameter evaluated in our study, i.e., non-specific antibody binding sites blocking with BSA, yielded similar results to pepsin digestion, demonstrating an observable effect on primary antibody staining intensity, while having no effect on Hoechst 33342 fluorescence. The latter is consistent with earlier reports indicating that Hoechst 33342 exhibits a low propensity for nonspecific interactions with cellular and extracellular components, resulting in stable nuclear labeling regardless of blocking conditions [16]. Blocking of nonspecific antigen binding sites is a standard and essential step in immunostaining protocols to enhance signal specificity and reduce background. Among protein-based blocking agents, BSA is one of the most frequently used due to its chemical stability, availability, and effectiveness. BSA is a neutral globular protein that adsorbs to free reactive sites on the substrates and tissue components, thereby preventing nonspecific interactions of antibodies with charged or hydrophobic surfaces [13,17,18]. In addition, BSA has been reported to reduce electrostatic and hydrophobic antibody binding, which is particularly relevant in protein-rich or highly charged biological matrices.

In the present study, no differences or increases in AF 488 signal intensity were observed following 45 min compared to 30 min BSA incubation. Notably, smaller pellets showed no difference in AF 488 signal intensity between the two incubation times, whereas larger pellets exhibited increased AF 488 signal following prolonged BSA incubation. This lack of change in smaller pellets, together with the signal increase observed in larger ones, suggests that a 45-min BSA incubation enhances the binding efficiency of the AF 488-conjugated anti-type II collagen antibody primarily in larger pellets. In theory, shorter blocking durations might be expected to yield higher fluorescence signals due to increased nonspecific antibody binding, potentially resulting in artificially elevated AF 488 intensity. However, this assumption does not fully account for the structural and compositional properties of chondrogenic pellets with a dense ECM. In such environments, antibody diffusion is limited, as antibodies may engage in transient, nonspecific interactions with ECM components before reaching their target epitopes [14]. Extended BSA incubation likely mitigates this diffusion limitation by more thoroughly saturating nonspecific binding sites within ECM. This enhanced blocking reduces transient antibody–matrix interactions, thereby facilitating deeper and more uniform antibody penetration. As a result, epitope accessibility is improved, leading to increased specific binding of the antibody and a corresponding enhancement of the AF 488 signal. Consistent with our findings, previous studies have shown that optimized blocking conditions improve antibody penetration and signal specificity in dense tissue and hydrogel-based systems [13,18].

Interestingly, the next tested parameter, i.e., the cell membrane permeabilization with Triton X-100, resulted in enhanced staining with both AF 488 and Hoechst 33342. Triton X-100 is a commonly used membrane–permeabilizing detergent that non-selectively extracts proteins in immunofluorescence protocols [19]. In principle, the permeabilization should not be required in our study, as collagen is synthesized and secreted by chondrocytes into the ECM [20]. Moreover, Hoechst 33342 is a small DNA intercalating dye that readily enters nuclei without membrane disruption [14,16]. However, increased AF 488 signal in permeabilized samples may be explained by intracellular labeling of procollagen, which is synthesized within the cell prior to secretion. Triton X-100 likely facilitates antibody access to intracellular procollagen, thereby increasing overall signal intensity [19]. Similarly, reduced Hoechst 33342 signal in the absence of Triton X-100 may reflect its limited membrane permeability, whereas permeabilization enables faster nuclear access and greater dye intercalation within the Hoechst 33342 staining time.

During staining of one of the control samples, structural disruption occurred during the Triton X-100 permeabilization step. A plausible explanation is that the ECM of controls (no-TGF-β1 controls) is mechanically less robust than that of treated samples, i.e., pellets treated with chondrogenic media [21,22,23], rendering it more susceptible to enzyme- and detergent-induced weakening and mechanical stress [24]. As Triton X-100 is used in multiple steps of the protocol, reducing exposure time or omitting selected steps may help minimize such disruption in pellets with less dense ECM [21,22,23,24]. Overall, the current protocol may be overly aggressive for non-chondrogenic or weakly organized matrix tissues, potentially contributing to the observed separation.

Next parameter tested, i.e., the nuclear staining using Hoechst 33342 incubation resulted in no substantial improvement of staining efficiency when incubation time was prolonged from 20 to 60 min, based on size-adjusted fluorescence intensity values, suggesting that dye penetration likely reaches an effective equilibrium within 20 min under the tested conditions. This is consistent with the notion that diffusion of small-molecule dyes, such as Hoechst 33342, into 3D models is relatively rapid and may not benefit from prolonged exposure time [14,16]. Overall, these findings suggest that increasing the incubation time does not improve nuclear staining efficiency and that a 20-min incubation is sufficient for reliable staining of pellets while minimizing the experimental time.

Finally, we evaluated the optimized immunofluorescence protocol for its ability to discriminate type II collagen content in pellets undergoing chondrogenic differentiation compared with no-TGF-β1controls cultured in the absence of chondrogenic induction. The size-adjusted AF 488 fluorescence intensity ranged from approximately no change to 3-fold higher in the treated samples than in the no-TGF-β1 controls, indicating a substantially enhanced deposition of type II collagen in response to chondrogenic stimulation. Increased type II collagen production is a well-established hallmark of differentiation and reflects the acquisition and maintenance of a chondrocyte-like extracellular matrix phenotype [5,6,7,9,11,25,26]. However, some exceptions were observed, particularly among chondrocyte samples (and also one primary MSC donor), where AF 488 fluorescence intensities were comparable between treated and no-TGF-β1 controls. One possible explanation is the spontaneous production of type II collagen in 3D chondrocyte cultures, which may diminish the detectable differences between treated and control groups. In contrast, the negative control (fibroblast cell line) displayed AFv488 fluorescence intensities comparable to background autofluorescence in both treated and no-TGF-β1 controls, thereby supporting the specificity of the detected signal. Taken together, these results demonstrate that this staining and detection protocol can detect chondrogenically differentiated samples in in vitro 3D settings. We acknowledge several limitations of our study. First, instead of pellet area, volume would be a more appropriate parameter for a true 3D normalization. However, the decision to use the area was based on technical limitations of the imaging system used. The full volumetric quantification was not feasible in our experimental setup due to limited light penetration and incomplete Z-stack acquisition across the entire pellet. Instead, we acquired Z-stacks only through regions where sufficient signal could be detected and generated a composite image for each pellet. Second, the use of the same AF 488 channel for both ROI definition and fluorescence quantification could, in principle, introduce bias. However, in our experimental setup, the baseline autofluorescence/background signal in the AF 488 channel was consistently sufficient and largely corresponded with the observed pellet boundaries in the brightfield image. This allowed us to reliably define pellet boundaries in both treated and no-TGF-β1 controls; hence, the pellet’s ROIs could be reliably delineated independently of differences in collagen II staining intensities. Brightfield images in our experimental setup demonstrated heterogeneous intensity distribution and lower segmentation consistency. Hence, they were used only for morphological assessment and not for the automated ROI generation. Third, a limited number of biological replicates, as the pellets were derived from only 5 donors. However, since the aim of our study was primarily optimization of the analysis, a lower number of donors was intentionally selected to minimize donor-to-donor variability and to enable a more controlled and focused assessment of the methodological parameters. Fourth, even though our protocol enables reliable visualization of chondrogenic features, we recognize that full biological validation requires complementary assays such as glycosaminoglycan content or gene expression analysis for key chondrogenic markers (e.g., *COL2A1*, *ACAN*). Last, further work is required to establish throughput capacity, reproducibility, and robustness across larger-scale experiments. Although this study is primarily methodological, it systematically evaluates key immunofluorescence parameters to establish a high-content imaging workflow for the quantitative assessment of type II collagen–rich neocartilage formation, providing a valuable tool for future studies investigating parameter-dependent effects on chondrogenesis, including collagen expression.

## 5. Conclusions

In conclusion, this study establishes immunofluorescence staining and a high-content imaging workflow for the quantitative assessment of 3D chondrogenesis of primary cells. Through systematic optimization of key experimental parameters, including staining platform, enzymatic matrix digestion, non-specific sites blocking, membrane permeabilization, and nuclear staining, we identified conditions that enable efficient antibody penetration, high signal specificity, and preservation of pellet morphology. Controlled pepsin-mediated matrix digestion and extended BSA blocking emerged as particularly critical determinants for achieving homogeneous and reliable detection of type II collagen within dense, ECM-rich pellets.

Importantly, coupling the optimized staining protocol with automated high-content non-confocal imaging allows rapid, quantitative, and scalable analysis of large pellet cohorts while accounting for pellet size–dependent effects through size-adjustment strategies. Within the scope of this study, the workflow enables discrimination between type II collagen-rich pellets and their controls, demonstrating its potential for comparative analyses across experimental conditions, tissue sources, and donor-derived primary cells.

Overall, the presented method addresses a key methodological gap in 3D cartilage tissue analytics by providing a fast and high-content imaging workflow for evaluating the chondrogenic potential in vitro. As such, it represents a practical tool for cartilage tissue engineering research and offers clear translational relevance for potency assessment, quality control, and early-stage screening of cell-based ATMPs targeting cartilage repair.

## Figures and Tables

**Table 1:** Parameters used for the optimization of the immunostaining protocol.

Sample (Donor) ID	Staining Platform (T/IP)	Chondrogenic Treatment (Tx/Ctl)	Pepsin Digestion (min)	BSA Blocking (min)	Triton X-100 (Yes/No)	Hoechst 33342 Exposure (min)	Type II Collagen Antibody (Yes/No)
1(A)	T	Tx	no	no	no	20	yes
2(A)	T	Tx	no	45	yes	20	yes
3(A)	T	Tx	10	45	yes	20	yes
4(A)	T	Tx	20	45	yes	20	yes
5(A)	T	Tx	30	45	yes	20	yes
6(A)	IP	Tx	no	no	no	no	no
7(B)	IP	Tx	20	45	yes	no	no
C1(B)	IP	Ctl	20	45	yes	no	no
8(B)	IP	Tx	20	45	yes	20	no
C2(B)	IP	Ctl	20	45	yes	20	no
9(A)	IP	Tx	10	45	yes	20	yes
10(C)	IP	Tx	20	30	yes	20	yes
11(C)	IP	Tx	20	45	yes	60	yes
12(C)	IP	Tx	20	45	yes	60	yes
13(C)	IP	Tx	20	30	yes	60	yes
14(C)	IP	Tx	20	30	yes	60	yes
15(A)	IP	Tx	20	45	no	20	yes
16(C)	IP	Tx	20	45	yes	20	yes
C3(C)	IP	Ctl	20	45	yes	20	yes
17(B)	IP	Tx	20	45	yes	20	yes
18(B)	IP	Tx	20	45	yes	20	yes
C4(B)	IP	Ctl	20	45	yes	20	yes
C5(B)	IP	Ctl	20	45	yes	20	yes
19(B)	IP	Tx	20	45	yes	20	yes
20(B)	IP	Tx	20	45	yes	20	yes
C6(B)	IP	Ctl	20	45	yes	20	yes
C7(B)	IP	Ctl	20	45	yes	20	yes
21(D)	IP	Tx	20	45	yes	20	yes
22(D)	IP	Tx	20	45	yes	20	yes
C8(D)	IP	Ctl	20	45	yes	20	yes
C9(D)	IP	Ctl	20	45	yes	20	yes
23(E)	IP	Tx	20	45	yes	20	yes
24(E)	IP	Tx	20	45	yes	20	yes
C10(E)	IP	Ctl	20	45	yes	20	yes
C11(E)	IP	Ctl	20	45	yes	20	yes
25(F)	IP	Tx	20	45	yes	20	yes
C12(F)	IP	Ctl	20	45	yes	20	yes

Sample (donor) identification (ID): samples 1–23 (donors A–F) represent pellets treated with TGF-β1; samples C1–C10 (donors A–F) represent no-TGF-β1 controls. T, 15 mL conical tube; IP, imaging plate; Tx, TGF-β1-treated pellets; Ctl, no-TGF-β1 controls; BSA, bovine serum albumin.

**Table 2:** Analysis of AF 488 fluorescence in samples used for optimization of the staining platform.

			AF 488		
Sample (Donor) ID	Staining Platform (T/IP)	Pellet Area [mm^2^]	Intensity [a.u.]	Size-Adjusted Intensity [a.u. × mm^2^]	CV [%]
3(A)	T	0.63	8100 ± 4200	5100 ± 2600	51.8
9(A)	IP	0.88	7800 ± 3800	6900 ± 3300	48.2

Values for fluorescent intensities of Alexa Fluor (AF) 488 are shown as mean ± standard deviation (SD) and coefficient of variation (CV). T, 15 mL conical tube; IP, imaging plate; a.u., arbitrary units.

**Table 3:** Analysis of AF 488 fluorescence in samples used for optimization of pepsin treatment.

			AF 488		
Sample (Donor) ID	Pepsin Treatment [min]	Pellet Area [mm^2^]	Intensity [a.u.]	Size-Adjusted Intensity [a.u. × mm^2^]	CV [%]
1(A)	no-pepsin control	0.86	5300 ± 2600	4500 ± 2200	49.6
2(A)	no-pepsin control	0.99	5100 ± 2300	5100 ± 2300	45.7
3(A)	10	0.63	8100 ± 4200	5100 ± 2600	51.8
4(A)	20	0.87	6400 ± 2900	5600 ± 2500	45.0
5(A)	30	0.80	6500 ± 3200	5200 ± 2600	49.9

Values for fluorescent intensities of Alexa Fluor (AF) 488 are shown as mean ± standard deviation (SD) and coefficient of variation (CV); a.u., arbitrary units.

**Table 4:** Analysis of AF 488 fluorescence in samples used for optimization of BSA incubation time.

			AF 488		
Sample (Donor) ID	BSA Incubation [min]	Pellet Area [mm^2^]	Intensity [a.u.]	Size-Adjusted Intensity [a.u. × mm^2^]	CV [%]
16(C)	45	0.83	9200 ± 4900	7600 ± 4100	53.3
10(C)	30	0.85	5900 ± 2800	5000 ± 2400	47.9
11(C)	45	0.42	11,800 ± 3500	4900 ± 1500	29.7
12(C)	45	0.47	10,400 ± 2800	4900 ± 1300	26.8
13(C)	30	0.52	8300 ± 3700	4300 ± 1900	44.3
14(C)	30	0.59	8400 ± 3700	5000 ± 2200	44.0

Values of fluorescent intensities for Alexa Fluor (AF) 488 are shown as mean ± standard deviation (SD) and coefficient of variation (CV). a.u., arbitrary units.

**Table 5:** Analysis of AF 488 fluorescence in samples used for optimization of permeabilization.

			AF 488		
Sample (Donor) ID	Triton X-100	Pellet Area [mm^2^]	Intensity [a.u.]	Size-Adjusted Intensity [a.u. × mm^2^]	CV [%]
15(A)	no Triton X-100 control	0.82	6500 ± 2500	5400 ± 2100	38.1
16(C)	with Triton X-100	0.83	9200 ± 4900	7600 ± 4100	53.3

Values for fluorescent intensities of Alexa Fluor (AF) 488 are shown as mean ± standard deviation (SD) and coefficient of variation (CV). a.u., arbitrary units.

**Table 6:** Analysis of fluorescence in samples used for optimization of Hoechst staining time.

			AF 488			Hoechst 33342		
Sample (Donor) ID	Hoechst 33342 [min]	Pellet Area [mm^2^]	Intensity [a.u.]	Size-Adjusted Intensity [a.u. × mm^2^]	CV [%]	Intensity	Size-Adjusted Intensity	CV [%]
10(C)	20	0.85	5900 ± 2800	5000 ± 2400	47.9	1730 ± 500	1470 ± 430	28.9
13(C)	60	0.52	8300 ± 3700	4300 ± 1900	44.3	2190 ± 1160	1150 ± 610	53.2
14(C)	60	0.59	8400 ± 3700	5000 ± 2200	44.0	3380 ± 1040	2010 ± 620	30.9
16(C)	20	0.83	9200 ± 4900	7600 ± 4100	53.3	1770 ± 500	1470 ± 410	28.1
11(C)	60	0.42	11,800 ± 3500	4900 ± 1500	29.7	3430 ± 850	1440 ± 360	24.8
12(C)	60	0.47	10,400 ± 2800	4900 ± 1300	26.8	2440 ± 770	1140 ± 360	31.5

Values of fluorescent intensities for Alexa Fluor (AF) 488 and Hoechst 33342 are shown as mean ± standard deviation (SD) and coefficient of variation (CV). a.u., arbitrary units.

**Table 7:** Analysis of AF488 fluorescence in samples undergoing chondrogenic differentiation.

			AF 488		
Sample (Donor) ID	Chondrogenic Treatment (Tx/Ctl)	Pellet Area [mm^2^]	Intensity [a.u.]	Size-Adjusted Intensity [a.u. × mm^2^]	CV [%]
6(A)	Tx	0.64	4400 ± 800	2800 ± 500	18.0
7(B)	Tx	0.45	2200 ± 800	1000 ± 400	24.9
C1(B)	Ctl	0.44	2100 ± 700	900 ± 300	25.5
8(B)	Tx	0.44	2400 ± 900	1000 ± 400	29.7
C2(B)	Ctl	0.43	2100 ± 700	900 ± 300	39.1
16(C)	Tx	0.83	9200 ± 4900	7600 ± 4100	53.3
C3(C)	Ctl	0.88	3400 ± 1700	3000 ± 1500	50.9
17(B)	Tx	0.43	6200 ± 1000	2600 ± 400	18.8
18(B)	Tx	0.44	5200 ± 500	2300 ± 200	20.4
C4(B)	Ctl	0.56	4000 ± 1600	2300 ± 900	30.0
C5(B)	Ctl	0.43	4600 ± 1600	2000 ± 700	34.2
19(B)	Tx	0.76	6000 ± 2000	4600 ± 1500	33.6
20(B)	Tx	0.59	4700 ± 600	2800 ± 400	13.6
C6(B)	Ctl	0.64	4300 ± 1100	2800 ± 700	26.1
C7(B)	Ctl	0.62	4500 ± 1200	2800 ± 700	25.6
21(D)	Tx	0.49	8500 ± 2900	4200 ± 1400	33.5
22(D)	Tx	0.67	8900 ± 3400	5900 ± 2200	37.7
C8(D)	Ctl	0.82	8000 ± 3200	6600 ± 2700	40.5
C9(D)	Ctl	0.67	7300 ± 2900	4900 ± 1900	39.1
23(E)	Tx	0.77	5800 ± 1800	4400 ± 1400	31.6
24(E)	Tx	0.57	7100 ± 2900	4000 ± 1700	41.1
C10(E)	Ctl	0.70	5100 ± 1300	3600 ± 900	25.2
C11(E)	Ctl	0.69	5100 ± 1400	3600 ± 900	26.3
25(F)	Tx	0.34	4300 ± 1200	1400 ± 400	27.7
C12(F)	Ctl	0.33	3300 ± 700	1100 ± 200	20.4

Values for fluorescent intensities of Alexa Fluor (AF) 488 are shown as mean ± standard deviation (SD) and coefficient of variation (CV). Sample (donor) identification (ID): samples 1–23 (donors A–F) represent pellets treated with TGF-β1; samples C1–C10 (donors A–F) represent no-TGF-β1 controls. Tx, TGF-β1-treated pellets; Ctl, no-TGF-β1 controls; a.u., arbitrary units.

## Data Availability

The original data presented in the study are openly available in the Repository of the University of Ljubljana at https://repozitorij.uni-lj.si/IzpisGradiva.php?id=182207&lang=eng (accessed on 25 May 2026).

## Supplementary Materials

The following supporting information can be downloaded at: https://www.mdpi.com/article/10.3390/biomedicines14071496/s1, Supplementary Figure S1: Individual and merged channels. Supplementary Table S1. Analysis of Hoechst 33342 fluorescence in samples used for optimization of the staining platform. Supplementary Table S2. Analysis of Hoechst 33342 fluorescence in samples used for optimization of pepsin treatment. Supplementary Table S3. Analysis of Hoechst 33342 fluorescence in samples used for optimization of BSA incubation time. Supplementary Table S4. Analysis of Hoechst 33342 fluorescence in samples used for optimization of permeabilization. Supplementary Table S5. Quantification of Hoechst 33342 fluorescence intensity for assessment of Type II collagen immunodetection.

## Abbreviations

The following abbreviations are used in this manuscript:

ATMPs	Advanced therapies medicinal products
MSCs	Mesenchymal stem/stromal cells
OA	Osteoarthritis
2D	Two-dimensional
3D	Three-dimensional
PBS	Phosphate buffered saline
BSA	Bovine serum albumin
ECM	Extracellular matrix
AF 488	Alexa Fluor 488
