# Supplementary material for: High-Content Analysis of 3D Chondrogenic Pellets Derived from Primary Cells In Vitro
Source: Biomedicines. 2026 Jul 1;14(7):1496. doi: 10.3390/biomedicines14071496 (PMC13403663; doi:10.3390/biomedicines14071496)
Supplement: Supplementary file 1 [file biomedicines-14-01496-s001.zip › biomedicines-4300578-supplementary.pdf]

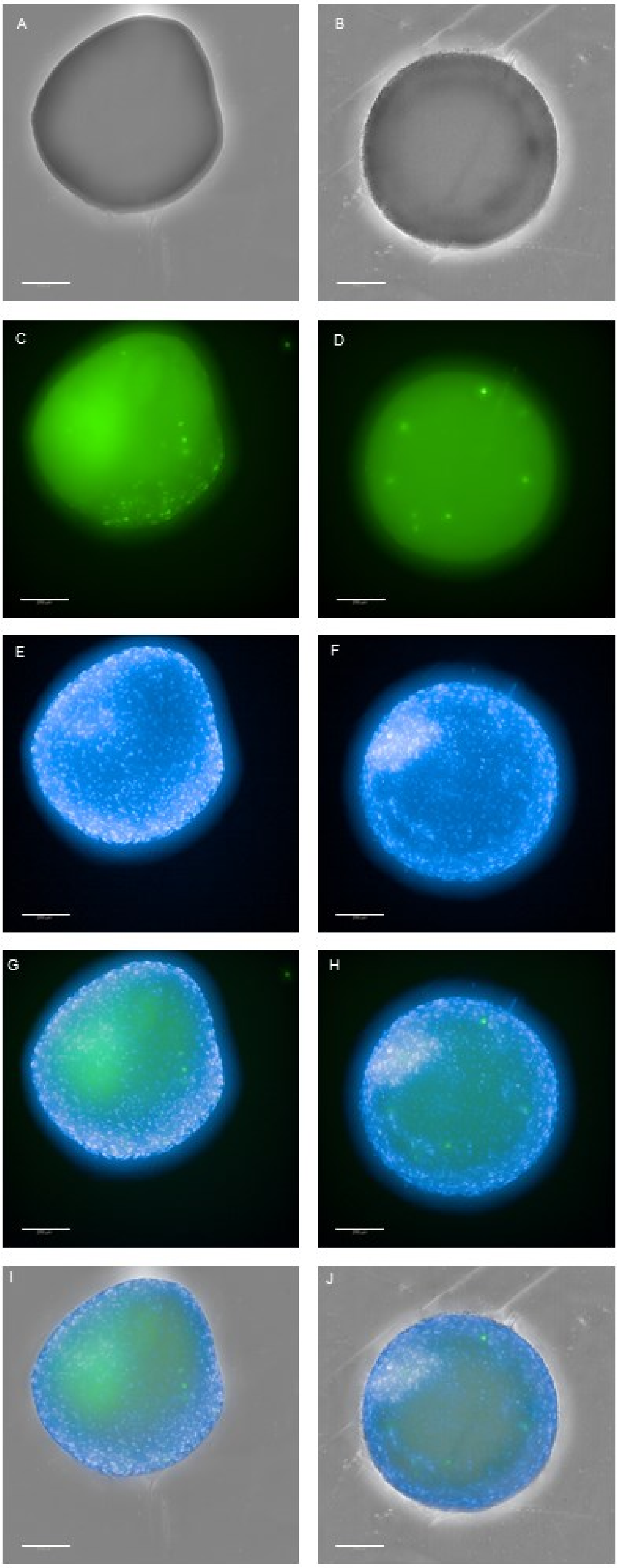

Supplementary Figure S1. Individual and merged channels. Representative images of primary MSCs (donor E, passage 3) showing individual brightfield (A, B) and fluorescence channels (AF488 in C, D; Hoechst 33342 in E, F), along with merged images (AF488 and Hoechst 33342 in G, H; all channels in I, J). Panels A, C, E, G, and I correspond to chondrogenically treated cell pellets, whereas panels B, D, F, H, and J show untreated (no-TGF-β1) controls.

Supplementary Table S1. Analysis of Hoechst 33342 fluorescence in samples used for optimization of the staining platform.

			Hoechst 33342		
**Sample (Donor) ID**	**Staining platform (T/IP)**	**Pellet area [mm²]**	**Intensity [a.u.]**	**Size-adjusted Intensity [a.u.×mm²]**	**CV [%]**
3(A)	T	0.63	670 ± 140	420 ± 90	21.1
9(A)	IP	0.88	1070 ± 420	950 ± 370	39.1

Values for fluorescent intensities of Hoechst 33342 are shown as mean ± standard deviation (SD) and coefficient of variation (CV). T, 15 mL conical tube; IP, imaging plate. a.u., arbitrary units.

Supplementary Table S2. Analysis of Hoechst 33342 fluorescence in samples used for optimization of pepsin treatment.

			Hoechst 33342		
**Sample (Donor) ID**	**Pepsin treatment [min]**	**Pellet area [mm^2]**	**Intensity [a.u.]**	**Size-adjusted Intensity [a.u.×mm^2]**	**CV [%]**
1(A)	no-pepsin control	0.86	720 ± 230	620 ± 200	32.4
2(A)	no-pepsin control	0.99	600 ± 170	600 ± 170	28.6
3(A)	10	0.63	670 ± 140	420 ± 90	21.1
4(A)	20	0.87	690 ± 190	600 ± 160	26.9
5(A)	30	0.80	660 ± 200	530 ± 160	30.0

Values for fluorescent intensities of Hoechst 33342 are shown as mean ± standard deviation (SD) and coefficient of variation (CV). a.u., arbitrary units.

Supplementary Table S3. Analysis of Hoechst 33342 fluorescence in samples used for optimization of BSA incubation time.

			Hoechst 33342		
**Sample (Donor) ID**	**BSA incubation [min]**	**Pellet area [mm²]**	**Intensity [a.u.]**	**Size-adjusted Intensity [a.u.×mm²]**	**CV [%]**
16(C)	45	0.83	1770 ± 500	1470 ± 410	28.1
10(C)	30	0.85	1730 ± 500	1470 ± 430	28.9
11(C)	45	0.42	3430 ± 850	1440 ± 360	24.8
12(C)	45	0.47	2440 ± 770	1140 ± 360	31.5
13(C)	30	0.52	2190 ± 1160	1150 ± 610	53.2
14(C)	30	0.59	3380 ± 1040	2010 ± 620	30.9

Values for fluorescent intensities of Hoechst 33342 are shown as mean ± standard deviation (SD) and coefficient of variation (CV). a.u., arbitrary units.

Supplementary Table S4. Analysis of Hoechst 33342 fluorescence in samples used for optimization of permeabilization.

			Hoechst 33342		
**Sample (Donor) ID**	**Triton X-100**	**Pellet area [mm²]**	**Intensity [a.u.]**	**Size-adjusted Intensity [a.u.×mm²]**	**CV [%]**
15(A)	no Triton X-100 control	0.82	1500 ± 790	1240 ± 650	52.3
16(C)	with Triton X-100	0.83	1800 ± 500	1470 ± 410	28.1

Values for fluorescent intensities of Hoechst 33342 are shown as mean ± standard deviation (SD) and coefficient of variation (CV). a.u., arbitrary units.

Supplementary Table S5. Quantification of Hoechst 33342 fluorescence intensity for assessment of Type II collagen immunodetection.

			Hoechst 33342		
**Sample (Donor) ID**	**Chondrogenic Treatment (Tx/Ctl)**	**Pellet area [mm²]**	**Intensity [a.u.]**	**Size-adjusted Intensity [a.u.×mm²]**	**CV [%]**
6(A)	Tx	0.64	240 ± 30	160 ± 20	12.6
7(B)	Tx	0.45	540 ± 160	240 ± 70	30.0
C1(B)	Ctl	0.44	350 ± 80	150 ± 40	23.1
8(B)	Tx	0.44	8980 ± 1830	3970 ± 810	20.4
C2(B)	Ctl	0.43	10390 ± 1950	4450 ± 840	18.8
16(C)	Tx	0.83	1770 ± 500	1470 ± 410	28.1
C3(C)	Ctl	0.88	2500 ± 1220	2200 ± 1070	48.7
17(B)	Tx	0.43	7910 ± 1970	3370 ± 840	24.9
18(B)	Tx	0.44	7220 ± 1840	3160 ± 810	25.5
C4(B)	Ctl	0.56	9600 ± 2850	5380 ± 1600	29.7
C5(B)	Ctl	0.43	8230 ± 3220	3530 ± 1380	39.1
19(B)	Tx	0.76	5240 ± 2780	3960 ± 2100	53.2
20(B)	Tx	0.59	8200 ± 1340	4840 ± 790	16.3
C6(B)	Ctl	0.64	6210 ± 2790	3990 ± 1790	45.0
C7(B)	Ctl	0.62	5690 ± 2790	3530 ± 1730	49.0
21(D)	Tx	0.49	36700 ± 1690	3320 ± 830	25.1
22(D)	Tx	0.67	7600 ± 2730	5070 ± 1820	35.9
C8(D)	Ctl	0.82	10700 ± 3920	8790 ± 3220	36.6
C9(D)	Ctl	0.67	9550 ± 2940	6400 ± 1970	30.8
23(E)	Tx	0.77	3010 ± 970	2300 ± 740	32.2
24(E)	Tx	0.57	2490 ± 710	1420 ± 400	28.3
C10(E)	Ctl	0.70	3270 ± 1080	2270 ± 750	33.2
C11(E)	Ctl	0.69	2960 ± 780	2050 ± 540	26.4
25(F)	Tx	0.34	3160 ± 940	1070 ± 320	29.9
C12(F)	Ctl	0.33	2820 ± 1250	930 ± 410	44.2

Values for fluorescent intensities of Hoechst 33342 are shown as mean ± standard deviation (SD) and coefficient of variation (CV). Sample (donor) identification (ID): samples 1-23 (donors A-F) represent pellets treated with TGF-β1; samples C1-C10 (donors A-F) represent no-TGF-β1 controls. Tx, TGF-β1-treated pellets; Ctl, no-TGF-β1 controls. a.u., arbitrary units.
